# Bioelectrical Impedance Analysis for the Assessment of Body Composition in Sarcopenia and Type 2 Diabetes

**DOI:** 10.3390/nu14091864

**Published:** 2022-04-29

**Authors:** Stefano Sbrignadello, Christian Göbl, Andrea Tura

**Affiliations:** 1 CNR Institute of Neuroscience, 35127 Padova, Italy; stefano.sbrignadello@cnr.it; 2 Department of Obstetrics and Gynaecology, Medical University of Vienna, 1090 Vienna, Austria; christian.goebl@meduniwien.ac.at

**Keywords:** sarcopenia, type 2 diabetes, bioelectrical impedance analysis, body composition, skeletal muscle mass, appendicular muscle mass

## Abstract

Sarcopenia is emerging as a severe complication in type 2 diabetes (T2DM). On the other hand, it has been documented that nutritional aspects, such as insufficient protein or total energy intake, increase sarcopenia risk. The analysis of body composition is a relevant approach to assess nutritional status, and different techniques are available. Among such techniques, bioelectrical impedance analysis (BIA) is particularly interesting, since it is non-invasive, simple, and less expensive than the other techniques. Therefore, we conducted a review study to analyze the studies using BIA for body composition analysis in T2DM patients with sarcopenia or at risk of catching it. Revised studies have provided important information concerning relationships between body composition parameters (mainly muscle mass) and other aspects of T2DM patients’ conditions, including different comorbidities, and information on how to avoid muscle mass deterioration. Such relevant findings suggest that BIA can be considered appropriate for body composition analysis in T2DM complicated by sarcopenia/muscle loss. The wide size of the patients’ cohort in many studies confirms that BIA is convenient for clinical applications. However, studies with a specific focus on the validation of BIA, in the peculiar population of patients with T2DM complicated by sarcopenia, should be considered.

## 1. Introduction

Sarcopenia is a syndrome characterized by low muscle mass, and either low muscle strength or low physical performance. The interest in the study of this syndrome has progressively increased, since it has become evident that sarcopenia not only determines poor quality of life, but also increases the risk for physical disability or even death [1,2,3,4]. In addition, it has been established that sarcopenia often coexists with typically chronic diseases or disorders, such as hypertension, obesity, and type 2 diabetes (T2DM) [5]. In fact, sarcopenia is emerging as a further severe complication in T2DM, in addition to those already well known, such as cardiovascular diseases [6]. In T2DM, the core pathophysiologic defects are insulin resistance in the muscle and in the liver, and pancreatic beta-cell dysfunction. However, it has been recognized that other factors play a relevant role in T2DM, especially accelerated lipolysis, gastrointestinal incretin hormones deficiency/resistance, hyperglucagonemia, increased glucose reabsorption, and brain insulin resistance (i.e., overall, the “Ominous Octet” [7]). Notably, these factors often present a common trait, that is, some degree of inflammatory condition. Inflammation, indeed, appears one of the factors which links T2DM and sarcopenia [8].

On the other hand, it has been documented that nutritional aspects, such as insufficient protein or total energy intake, or gastrointestinal diseases (malabsorption), increase the risk for sarcopenia or further worsen it after its onset [9,10], and of course, nutrition is particularly relevant in subjects with concomitant sarcopenia and T2DM [11,12]. In this context, the analysis of body composition is a relevant approach, as it helps to assess the overall nutritional status [13,14]. To this purpose, different techniques are available. Simple anthropometric measures, such as the body mass index (BMI), waist and hip circumference, or the waist-to-hip ratio, can be considered basic markers of body composition, whereas accurate and powerful techniques include dual-energy X-ray absorptiometry (DXA), computed tomography (CT), and magnetic resonance imaging (MRI) [14]. However, these refined techniques may not be optimal for application in the clinical routine, due to factors such as cost, time required for the examination, and possible burden to the patient. Thus, an alternative to those techniques is the bioelectrical impedance analysis (BIA) [14]. 

Of note, in sarcopenia, one aspect of body composition is particularly important, i.e., the assessment of skeletal muscle mass. Indeed, muscle mass quantification is indicated as one of the criterion for the diagnosis of sarcopenia, according to the guidelines of the European Working Group on Sarcopenia in Older People (EWGSOP) [15,16]. Precisely, low muscle mass is required for sarcopenia diagnosis, whereas one further condition is sufficient between either low muscle strength or, alternatively, impaired physical performance [15,16]. It has to be noted that other guidelines for sarcopenia diagnosis exist, such as those from the Asian Working Group for Sarcopenia [17,18], or those from the Japan Society of Hepatology [19]. However, all guidelines essentially agree on the role of muscle mass and strength in sarcopenia. Among the indicated techniques for body composition assessment (and specifically for muscle mass quantification), BIA appears particularly interesting, since in our opinion it is likely that they are the most “cost-effective”. In fact, BIA may be more accurate than possible approaches based only on anthropometric measures [20], especially when the balance between fat mass and fat-free mass is abnormal (as it can happen in sarcopenia). In contrast, when compared to the refined techniques (DXA, CT, MRI), accuracy may be somehow lower; however, BIA has the advantages previously indicated. In summary, BIA may be the best compromise between cost and simplicity; hence, it appears particularly adequate for use in the clinical routine. Of note, EWGSOP explicitly indicates BIA as an appropriate/acceptable technique for muscle mass quantification in the clinical practice [15,16]. Accordingly, BIA has been used in several studies related to sarcopenia, as summarized by some recent reviews [21,22]. BIA has also been used in several studies on people with diabetes, mainly type 2, but also gestational and type 1 diabetes (including Wang et al. [23], Bai et al. [24], and Arpaia et al. [25]). Furthermore, BIA has been used in several studies related to concomitant sarcopenia and type 2 diabetes. The aim of the current review is to summarize those studies, with focus on the usefulness (but also possible limitations) of BIA for the goals and outcomes of the analyzed studies. To our knowledge, this is the first review study focused on BIA in sarcopenia and diabetes.

## 2. Methodology for Scientific Literature Search

The search of scientific literature was performed in PubMed. Following testing of different search strings, we identified this final string: 

(bio-impedance[tw] OR bioimpedance[tw] OR ((bioelectric*[tw]) AND impedance[tw])) AND sarcopen*[tw] AND (diabet*[tw] OR prediabet*[tw] OR hyperglicem*[tw] OR hyperglycaem*[tw] OR (impaired[tw] AND glucose[tw])).

According to PubMed guidelines, “tw” (“text word”) searches all main fields of PubMed records, i.e., in article titles, abstracts, MeSH terms, plus some additional fields. The symbol “*” searches for all variations of a word root, e.g., sarcopenia and sarcopenic, etc.

Notably, to identify all possible articles of potential interest for our review, we searched for all possible variations for BIA indication, as well as all variations of type 2 diabetes. Indeed, for highest generalization, we also searched for prediabetes, hyperglycemia, or similar expressions, and we did not specify “type 2”, though this was the focus of our review.

**Figure 1:**
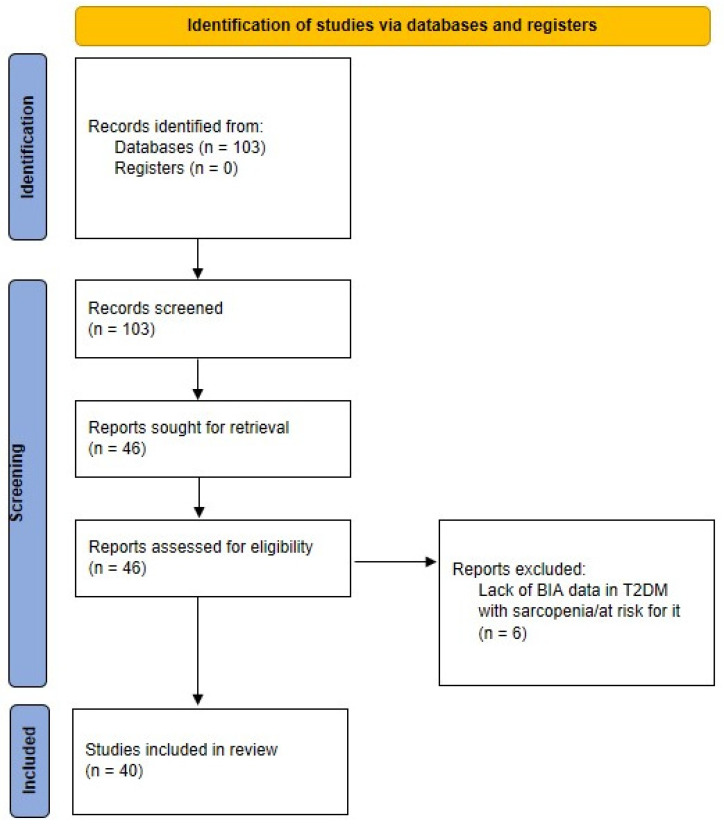
PRISMA flow diagram of the scientific literature search strategy.

The indicated search strategy yielded 103 items (last check: 4 March 2022). We therefore analyzed each item, and ended with a set of 40 articles as pertinent for our review. In fact, we selected those articles actually including BIA data (i.e., not only mentioning BIA), and, similarly, explicitly presenting data in T2DM patients with sarcopenia (or at risk for it). Our search process is schematized in Figure 1.

**Figure 2:**
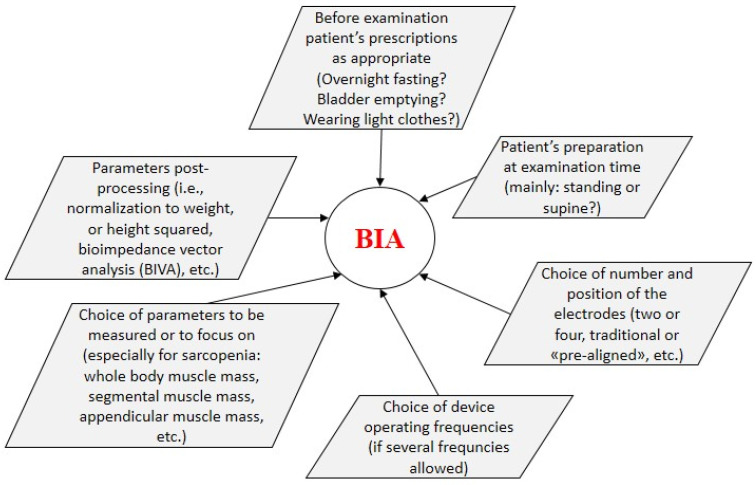
Methodological aspects related to BIA examination.

In the following sections, we summarize the main aspects of the selected studies, with special focus on BIA data and role. In each section, articles are presented in chronological order. Some synthetic information for each study are reported in Table 1. Some relevant methodological aspects related to BIA examination, explored when presenting the selected studies, are summarized in Figure 2.

## 3. BIA in Sarcopenia and T2DM: Cross-Sectional Studies

In the present section, we describe cross-sectional studies where BIA was applied to subjects with T2DM and sarcopenia (or at risk for it). We include here those studies where there is no additional information of nutritional type, whereas we will describe studies where body composition data are complemented with explicit nutritional information in the following section, such as diet habits, etc. The present section is organized in two subjections: the “early” studies (until 2019), and the recent studies (i.e., in the last years: since 2020 onwards).

### 3.1. “Early” Studies

The first study that we have identified as pertinent for our review was carried out in 2010, by Tajiri et al. [26]. Body composition was analyzed using BIA in 198 patients with T2DM, and the same number of healthy subjects were used for comparison, matched for gender, age, and BMI (107 men and 91 women in both groups). It is reported that BIA was performed by the INBODY720 device, without further details. The skeletal muscle amount (M) and its percentage related to body weight (M%) were assessed for the whole body and at isolated level (in each arm and leg). The fat mass (F) and the percentage for body weight (F%) were also estimated. Results showed that whole body M%, especially that of the lower limbs, was lower in T2DM compared to normal subjects. A progressive reduction of M% in the lower limbs was assigned to an increasing risk factor for cardiovascular disease. Regional sarcopenia (at lower limbs, named “leg sarcopenia”) was found in long-term diabetic patients with insufficient physical exercise.

Somehow, similar to the Tajiri’s study, in 2013, Buffa et al. [27] investigated the characteristics of body composition in elderly subjects with T2DM, compared with healthy controls matched for age and BMI. A specific type of BIA analysis, called bioelectrical impedance vector analysis, was applied. T2DM patients were 144 free-living subjects (84 women and 60 men), 60 to 84 years old, whereas healthy controls were 209 (116 women and 93 men). Bioelectrical impedance vector analysis helped to calculate body resistance (R) and reactance (Xc), measured with a single-frequency impedance analyzer (BIA 101, Akern srl, Florence, Italy), and standardized by height. The phase angle was calculated as arctan (Xc/R) and the impedance vector as (R^2^ + Xc^2^)^0.5^. Individual vectors of the bioelectrical values in the healthy controls were exploited to plot the so-called “tolerance ellipses”. The vector position in the plane was then assumed as an indicator of body composition, the minor axis indicating the cell mass, and major axis referring to the hydration status. It was found that, compared to healthy subjects, T2DM female patients showed lower resistance, and male patients showed higher reactance. It was concluded that T2DM patients showed bioelectrical abnormalities that can be related to smaller appendicular muscular area and lower extracellular/intracellular water ratio, and such abnormalities can be risk factors for sarcopenia. 

In 2016, the study by Rizzo et al. [28] investigated the effects on sarcopenic parameters of antidiabetic agents, i.e., dipeptidyl peptidase 4 inhibitors (DPP4-I) or sulphonylureas (following at least 24 months of treatment). A group of 80 elderly T2DM patients was studied (42 males and 38 females, 65 years or older with minimum 5-year since T2DM diagnosis). Body composition was assessed using a BIA Handy device (DS Medica, Milan, Italy). Bioimpedance was measured (coefficient of variation of 16%); this helped to estimate fat-free mass (FFM), fat mass (FM), the FFM/FM ratio, the total body water, and extracellular and intracellular water. Skeletal muscle mass (SMM) was calculated using a BIA-based equation, i.e., SMM = 0.401 × (height^2^/resistance) + (3.825 × gender) − (0.071 × age) + 5.102, with height in cm, resistance in ohm, and gender equal to 1 for men and 0 for women. The SMM index was then computed as 100 × SMM, normalized to height squared, and it was considered abnormal if lower than 8.87 kg/m^2^ in men and 6.42 kg/m^2^ in women. It was found that those treated with DPP4-I showed better sarcopenic parameters compared with those under sulphonylureas. Specifically, the DPP4-I group had greater muscle mass, and better physical performance and strength, as compared to the sulfonylureas-treated group. However, it was reported that it was currently not known whether the action mechanism of DPP4-I on sarcopenic parameters is direct or indirect.

In the same year, Hashimoto et al. [29] investigated the association between hepatic steatosis and the skeletal muscle mass index (SMI) in 145 Japanese T2DM patients (79 men and 66 women). The investigation was motivated by the fact that some previous studies revealed an association between sarcopenia and fatty liver diseases; however, specific analysis in T2DM patients had not been performed at that time. BIA was measured using a multifrequency impedance body composition analyzer (InBody 720, InBody Japan Inc., Tokyo, Japan). The SMI was defined as the skeletal muscle mass normalized to total body weight × 100. The visceral fat area was also assessed. Hepatic steatosis was evaluated with transient elastography. Results showed that the SMI was inversely associated with hepatic steatosis in T2DM men, likely due to the fact that reduced skeletal muscle mass is associated with insulin resistance; on the other hand, insulin resistance is typical of the hepatic steatosis and of the T2DM condition. In addition, the insulin-like growth factor 1 (IGF-1) was found decreased in patients with insulin resistance, and showed an important role on skeletal muscle mass.

In 2017, Tuzun et al. [30] assessed the relationship between bioimpedance measurements and metabolic parameters in T2DM patients, at possible risk for sarcopenia. A group of 359 patients aged less than 65 years was studied (81 men, 278 women). BIA analysis was performed using a TANITA 48M device (no further details provided). Body fat mass, total muscle mass, and appendicular muscle mass were derived using BIA. The skeletal muscle index and percentage were calculated as the appendicular muscle mass normalized by body height squared and by body weight, respectively. Total muscle index and percentage were similarly calculated from the total muscle mass. It was found that, after adjusting for age and gender, there was no relationship between muscle-related parameters and fasting plasma glucose, as well as triglycerides and LDL-cholesterol; however, there was direct correlation with C-peptide, and inverse correlation with HDL-cholesterol.

Several studies relevant for our review were performed in 2018 and 2019. In 2018, Osaka et al. [31] investigated the hypothesis that the ratio between serum creatinine and cystatin C (Cre/CysC) could be used as a surrogate marker for sarcopenia. A group of 285 patients with T2DM was studied (159 man, 126 women). BIA was measured using the InBody 720 (InBody Japan Inc., Tokyo, Japan). The skeletal muscle index (SMI) was calculated as the appendicular skeletal muscle mass divided by height squared. For sarcopenia definition, Japan Society of Hepatology guidelines were considered [19]; thus, sarcopenia was defined as an SMI of <7.0 kg/m^2^ in men and <5.7 kg/m^2^ in women, and handgrip strength was <26 kg/m^2^ in men and <18 kg/m^2^ in women. Results showed that 8.8% of the patients had sarcopenia. Authors suggested a cut-off value of 0.90 (higher value meaning higher sarcopenia risk) for the Cre/CysC ratio to detect sarcopenia, and concluded that the Cre/CysC ratio is usable as a simple screening tool to identify T2DM patients at high risk for sarcopenia, especially in patients also suffering for renal dysfunction. However, it was observed that possible causal relationship between sarcopenia and the Cre/CysC ratio needed further investigation.

In the 2018 study, Tuzun et al. [32] assessed the prevalence of sarcopenia in T2DM patients using different criteria based on BIA. In total, 295 patients were involved in the study (176 females, 119 males), with age ≥ 18 years and BMI ≥ 30 kg/m^2^. BIA was performed in all participants using TANITA-48M device (TANITA, Tokyo, Japan). BIA measurements consisted of total fat mass, total muscle mass, and sum of the appendicular muscle masses (ALM) of the four limbs. Total muscle mass was calculated with the same formula previously reported for the study of Rizzo et al. [28], and the skeletal muscle index was assessed as the total muscle mass divided by the body height squared. The body muscle ratio was assessed as the total muscle mass divided by the body weight. Body fat percentage was calculated as the ratio of total fat mass to body weight, multiplied by 100. For the skeletal muscle index and body muscle ratio, some cut-off values were identified based on the analysis of such parameters distribution in young healthy people. Thus, sarcopenia of Class 1 was considered for the skeletal muscle index in the 8.51–10.75 range for men and in the 5.76–6.75 range for women (units: kg/m^2^). Accordingly, Class 2 sarcopenia was defined for the skeletal muscle index, i.e., <8.50 in men and <5.75 in women. Alternatively, Class 1 sarcopenia was defined as the body muscle ratio in the 31.5–37.0 range for men and the 22.1–27.6 range for women (units: %), whereas Class 2 required a body muscle ratio of <31.5 in men and <22.1 in women. Class 1 sarcopenia was also defined as a ALM/BMI ratio of <0.789 in men and <0.512 in women (units: kg/kg/m^2^). Results showed that sarcopenia was determined in 40 males using the body muscle ratio, in 15 using the ALM/BMI ratio, and in 1 participant using the skeletal muscle index. In females, sarcopenia was found in 61 participants using the body muscle ratio, in 1 participant using the ALM/BMI ratio, and in none using the skeletal muscle index. It was concluded that the prevalence of sarcopenia is low in obese T2DM when the skeletal muscle index or the ALM/BMI ratio are used, and definitely higher when the body muscle ratio is used.

The study by Murai et al. [33] aimed to investigate the associations among visceral fat accumulation, skeletal muscle indices (mass, strength, and quality), and cardiovascular diseases in 183 hospitalized T2DM patients (126 men and 57 women, 33–88 years). The visceral fat area was measured using the EW-FA90 (Panasonic Corporation) instrument, while the muscle masses of the trunk, arms, and legs were measured using the InBody 720 (InBody Japan Inc., Tokyo, Japan) BIA device. The skeletal muscle index (SMI) was defined as the height-adjusted appendicular skeletal muscle mass, i.e., muscle mass of the arms and legs normalized to height squared. The grip strength was measured using a dynamometer, and muscle quality was calculated as the ratio of grip strength to the arm muscle mass, measured on both sides and then averaged. Several blood parameters possibly related to cardiovascular diseases were also measured, such as glucose, glycated hemoglobin (HbA1c), C-peptide, alanine transaminase, uric acid, cholesterol, triglycerides, creatinine, CRP, and brain natriuretic peptide. The prevalence of sarcopenia was defined by the criteria of the Asian Working Group [17]. Thus, cut-off values for the SMI were set to 7.0 kg/m^2^ for men and 5.7 kg/m^2^ for women. Cut-off values for grip strength were set to 26 kg for men and 18 kg for women. Sarcopenia was considered present when both the SMI and grip strength were below the respective cut-off values. Results showed that sarcopenia was present in 22% of the patients. Skeletal muscle mass was lower in the visceral fat accumulation group than in the other subjects’ group. Muscle quality was also significantly lower in patients with visceral fat accumulation. Furthermore, in the visceral fat accumulation group, patients with low muscle quality had higher prevalence of cardiovascular diseases, and longer duration of diabetes. Sex- and age-adjusted models showed significant association between low muscle quality and cardiovascular diseases in all subjects, but especially in patients with visceral fat accumulation. It was concluded that T2DM patients with visceral fat accumulation had low muscle quality, and patients with low muscle quality were more affected with cardiovascular diseases. 

In the 2018 study, Hashimoto et al. [34] investigated the association between sarcopenia and blood pressure parameters in elderly patients with T2DM. In total, 146 patients (86 men and 60 women), aged ≥ 65 years, were studied. Body fat mass, skeletal muscle mass, and appendicular muscle mass were obtained using the BIA InBody 720 (InBody Japan, Tokyo, Japan). The skeletal muscle mass index (SMI) was calculated as the appendicular muscle mass normalized to height squared. The definition of sarcopenia was based on the guidelines for sarcopenia of the Asian Working Group for Sarcopenia, requiring both the SMI and handgrip strength impairment [17]. The prevalence of sarcopenia was 14.4%. In patients with sarcopenia, the coefficient of variation of systolic blood pressure (SBP) was higher than that in patients without sarcopenia, although the average SBP was not different between sarcopenic and non-sarcopenic subjects. In addition, regression analysis showed that sarcopenia was associated with the coefficient of variation of SBP even after adjusting for covariates, whereas sarcopenia was not associated with the average SBP. It was concluded that sarcopenia is associated with blood pressure variability, but not with its absolute values. 

In 2019, Fukuoka et al. [35] investigated the prevalence of sarcopenia, its related factors, and the indicators of physical performance in elderly T2DM. A group of 267 patients (159 men, 108 women), aged > 65 years, was examined. Body composition was measured using InBody 770 (InBody, Japan Inc., Tokyo, Japan). The skeletal muscle mass index (SMI) was calculated by dividing the limb skeletal muscle mass by height squared, and a low SMI was defined as SMI < 7.0 kg/m^2^ in men and <5.7 kg/m^2^ in women, in agreement with previous studies, such as that by Hashimoto et al. [34] illustrated above. In addition to the SMI assessed by BIA, the grip strength and the usual gait speed were measured as indicators of physical performance. The prevalence of sarcopenia was 18.7%. Analyses showed that sarcopenia decreased as BMI increased, whereas sarcopenia tended to increase for higher body fat. In addition, sarcopenia was associated with the non-use of metformin and lower bone mineral content in men, as well as lower bone mineral content, lower serum levels of albumin, and older age in women. It was concluded that T2DM patients with high body fat percentage in addition to low BMI may develop sarcopenia.

The study by Oh et al. [36] in 2019 aimed to evaluate muscle mass, strength, and physical performance in subjects with T2DM and assess whether diabetic peripheral neuropathy was a significant risk factor for sarcopenia. In total, 170 patients were studied, aged 50 years and older (93 males, 77 females). Sarcopenia was diagnosed, according to the Asian Working Group for Sarcopenia criteria [17]. To this purpose, in addition to BIA, a handgrip test and a gait speed test over 4 m walking distance were performed in all patients. BIA was performed using the InBody 770 device (InBody, Seoul, Korea). Of note, in the article, it is specified that participants were asked to wear light clothes for BIA measurements. The parameter considered as the muscle mass index was the appendicular skeletal muscle mass divided by the height squared. Various examinations of neuropathy were also carried out, including both small- and large-fiber neuropathy. In addition, neuropathy was also evaluated using an appropriate questionnaire (Michigan Neuropathy Screening Instrument Questionnaire). It was found that sarcopenia prevalence was at 14.1%. The questionnaire scores were higher in patients with sarcopenia, although other neuropathy examination results were not significantly associated with sarcopenia. It was concluded that active screening for sarcopenia should be performed in subjects with diabetic peripheral neuropathy. 

A similar study was performed in the same year by Yasemin et al. [37], but with a larger number of patients. Indeed, 602 T2DM patients were studied, from 18 years onwards, but with average age of 60.2 years (244 men, 359 women). Of them, 512 had diabetic neuropathy. BIA was performed with patients in light clothes using the Tanita body composition analyzer (no further details reported). Absolute skeletal muscle mass was converted into the percentage skeletal muscle mass (muscle mass/body mass × 100) and termed as the skeletal muscle index (SMI). Those who had a SMI less than a −1 standard deviation of the average SMI in young adults aged 18–40 years were defined as having low muscle mass. Thus, based on this, the cut-off level for SMI was taken as 37% for men and 28% for women. The handgrip test was also performed (cut-off of 30 and 20 for men and women, respectively). Those who showed only reduced handgrip strength were categorized in the s-presarcopenia group, whereas patients with only muscle mass (volume) loss were categorized in the v-presarcopenia group; those who had both defects were defined as sarcopenic. Sarcopenia that was accompanied by obesity was defined as sarcopenic obesity. It was found that sarcopenia prevalence was higher in in patients with diabetic neuropathy than in those without it (24.7% vs. 8.9%). On the other hand, diabetic neuropathy prevalence was 80.2% in those who had normal muscle mass and strength, 84.4% in s-presarcopenic patients, 82.1% in v-presarcopenic patients, and 94.1% in sarcopenic patients. Of note, diabetic neuropathy prevalence reached 95.9% in sarcopenic obese patients. Thus, the study revealed a clear relation between sarcopenia and diabetic neuropathy.

The study by Su et al. [38] aimed to investigate the value of the skeletal-to-visceral ratio (SVR) in the prediction of non-alcoholic fatty liver disease (NAFLD) in patients with T2DM, possibly complicated by sarcopenia or sarcopenic obesity. In total, 445 T2DM patients were recruited (236 men, 209 women, aged 40–75 years). The lean body mass of arms and legs, the appendicular skeletal muscle mass (ASM, as a sum of the lean soft tissue masses in the arms and legs), and the visceral fat area (VFA) were calculated by BIA (InBody 720; Biospace, Land Seoul, Korea). Notably, the analyzer measured resistance at six frequencies (1, 5, 50, 250, 500 kHz, and 1 MHz) and reactance at three frequencies (5, 50, and 250 kHz). Of note, it was observed that good correlation was shown between VFA measured by BIA and that measured by abdominal computed tomography. SVR was calculated as ASM normalized to VFA, and assumed as an index of sarcopenic obesity. Hepatic steatosis for NAFLD diagnosis was assessed using the ultrasonic approach. Results showed that NAFLD prevalence increased with decreased SVR (significant differences were observed between the highest and lowest tertiles). Furthermore, SVR values in the lowest tertiles were independently associated with the presence of NAFLD in females. It was concluded that T2DM patients with lower SVR levels (who may also be sarcopenic) are associated with higher risks of developing NAFLD-related complications.

### 3.2. Recent Studies

In this subsection, we include those studies that we have defined as “recent”, that is, from 2020 onwards.

The study by Medeiros et al. [39] aimed to study patients on hemodialysis and determine the associations of sarcopenia with serum sclerostin concentrations, which is an osteoblast-inhibiting glycoprotein secreted mainly by osteocytes and regulated by hormonal changes and skeletal loading. A group of 92 hemodialysis patients (average age of 63.3 years) was studied—41 with T2DM (26 males, 15 females) and 51 without diabetes (32 males, 19 females). Multifrequency electric BIA (Biodynamics 310, Biodynamics Corporation, Shoreline, WA, USA) was performed immediately after hemodialysis, that is, with a weight considered to be “dry”. The fat-free mass index was calculated by dividing fat-free body mass by squared height. This was assumed as the skeletal muscle mass index. Values of 10.75 kg/m^2^ or less in men and 6.75 kg/m^2^ or less in women were considered to be low. Handgrip strength and physical performance tests were also performed for the diagnosis of sarcopenia. A low muscle mass index was identified in 65.2% of the individuals studied (76.7% were male and 36.7% diabetic), of which 10.9% were at the pre-sarcopenia stage, 23.9% had sarcopenia, and 30.4% had severe sarcopenia. Mean serum sclerostin was higher in men, in those individuals with the low muscle mass index, and in diabetic patients. After adjustments for potential confounders, high serum sclerostin was independently associated with the low muscle mass index and with presence of diabetes. It was concluded that serum sclerostin is directly related to diabetes and inversely related to muscle mass in hemodialysis patients.

The study by Seo et al. [40] aimed to evaluate the association between skeletal muscle mass and carotid atherosclerosis in men and women with T2DM. In fact, it was observed that sarcopenia was already known to lead to metabolic and vascular abnormalities; however, in T2DM patients, little was known regarding the independent relationship between skeletal muscle mass and atherosclerosis. In total, 8202 patients with T2DM were recruited (4156 men, 4046 women), with age ≥ 19 years (average age equal to 57.7 years). Body composition was assessed using a segmental multifrequency BIA device (InBody 4.0; Biospace, Seoul, Korea). The skeletal muscle mass (SMM) was recorded in kilograms, while the skeletal muscle mass index SMI was calculated by dividing the SMM by total body weight × 100. Both carotid arteries were examined using B-mode ultrasound (carotid atherosclerosis was defined by having a carotid plaque or mean carotid intima-media thickness ≥ 1.1 mm). It was found that among the entire population, 52.4% of subjects had carotid atherosclerosis, and the prevalence of carotid atherosclerosis increased with decreasing SMI quartiles for both sexes. In addition, in men, the risk of atherosclerosis increased linearly with decreasing SMI quartiles. It was concluded that low skeletal muscle mass was independently associated with the presence of carotid atherosclerosis in T2DM. 

In the study by Jung et al. [41], the main aim was to investigate the relationship of sarcopenia with microcirculatory function, as assessed by skin perfusion pressure (SPP), in T2DM patients. In total, 102 T2DM patients (average age 55.9 years, 63.7% men), who underwent both SPP measurements and BIA, were enrolled in the study. BIA was used to determine the appendicular skeletal muscle mass (ASM), which was calculated by summing the lean mass in the arms and legs, primarily representing skeletal muscle mass in the extremities (the used BIA device was however not indicated). Sarcopenia was defined as low muscle mass, based on ASM divided by height squared, with cut-off values of 7 kg/m^2^ in men and 5.7 kg/m^2^ in women. SPP was assessed using the laser Doppler technique. Participants were divided into two groups based on SPP (≤50 and >50 mm Hg), and the low SPP group was considered as having impaired microcirculation. Based on such definition, 13.7% of participants were diagnosed with impaired microcirculatory function. The prevalence of sarcopenia in all subjects was 11.8%; however, the percentage of patients with low SPP who had sarcopenia was more than triple that of patients with normal SPP. In addition, a positive correlation was found between SPP and appendicular muscle mass adjusted for height. Thus, results suggested that sarcopenia may be significantly associated with impaired microcirculation in patients with T2DM, though it was acknowledged that the relatively small number of patients required cautious interpretation of such findings. 

Somehow, the study by Seo et al. [42] had similar aims to the study by Su et al. [38]; however, it covered a higher number of subjects. Indeed, in Seo’s study, association between sarcopenia and NAFLD was investigated separately in men and women with T2DM, in a cohort of 4210 patients (2160 men, 2050 women, average age of 57.4 years). BIA (by InBody 4.0, InBody Co., Ltd., Seoul, Korea) was used to determine the appendicular skeletal muscle mass (ASM), calculated as the sum of the lean mass in arms and legs, similar to the study by Jung et al. [41] illustrated above. The skeletal muscle mass index (SMI) was then calculated as ASM normalized to body weight. Sarcopenia was defined as a gender-specific SMI value > 2 standard deviations below the mean for healthy young adults. NAFLD was defined as the presence of hepatic steatosis on ultrasonography with no other causes of chronic liver disease. It was found that 29.5% of the patients had sarcopenia, whereas 30.4% had NAFLD, and the prevalence of NAFLD was significantly higher in those with sarcopenia, both in men and in women. In addition, sarcopenia was significantly associated with higher risk of NAFLD in men, while the association was attenuated in women after adjusting for clinical risk factors. Thus, in men with T2DM, sarcopenia appears independently associated with NAFLD, which suggests that sarcopenia may be a risk factor for NAFLD in T2DM men.

The study by Low et al. [43] moved from the consideration that lower extremity skeletal muscle mass (LESM) in T2DM has been linked to several adverse clinical events; however, at the time of the study, it was not known whether it was associated with cognitive difficulties. Thus, the aim of the study was to investigate whether low LESM, possibly in parallel with a low upper extremity skeletal muscle mass (UESM) and a low total appendicular skeletal mass index (SMI), is associated with reduced cognitive function in people with T2DM. In total, 1235 T2DM patients were studied (641 males, 594 females, aged > 45 years). Body composition was assessed by tetrapolar multifrequency BIA device (InBody-S10; Biospace, Cerritos, CA, USA). Skeletal muscle masses in left and right lower limbs were added and divided by height squared to calculate LESM. UESM was calculated similarly by upper limbs masses. The skeletal muscle mass index (SMI) was calculated as the appendicular lean mass (mass of the four limbs) divided by height squared, which is also useful for the diagnosis of sarcopenia (which may share common underlying background with cognitive impairment). Cognitive function was assessed with appropriate tests, which helped to evaluate five domains of cognition (attention, language, visuospatial/constructional abilities, immediate and delayed memory); hence, an overall cognition score was derived from the five domains. Results showed that LESM, as well as UESM and SMI, were related to the cognition score; however, when examining the single cognition domains, such relationships were not always present. It was concluded that especially lower LESM may be a useful marker of possible co-occurring cognitive dysfunction.

The study by Lin et al. [44] analyzed the association of body composition with T2DM, with focus on sarcopenic obesity. Body composition was measured through multifrequency BIA (MF-BIA; InBody 770, Cerritos, CA, USA). BIA was conducted in individuals with T2DM, who were aged ≥ 18 years. In total, 2404 patients were analyzed (1275 men, 1129 women), in a wide span of age (2.2% in 18–<35 years range, 49.8% in 35–<65 range, 27.4% in 65–<75 range, and finally 20.6% aged > 75 years). Fat-free mass (FFM, i.e., muscles, bones, organs, and body fluids), body fat mass (BFM, as total weight—FFM), percent body fat (PBF, as body fat mass/body weight × 100), visceral fat area (VFA, i.e., the estimated area of fat surrounding internal organs in the abdomen), appendicular skeletal muscle mass (ASM), and skeletal muscle index (SMI, as ASM/height squared) were collected for the analyses. Low muscle mass was defined using the SMI (male: <7.0 kg/m^2^; female: <5.7 kg/ m^2^), and sarcopenic obesity was defined as low SMI plus high PBF (male: low SMI and PBF ≥ 25%; female: low SMI and PBF ≥ 30%). The prevalence of overall low muscle mass and sarcopenic obesity was 28.0% and 18.7%, respectively, which increased with age. Interestingly, the normal BMI group exhibited a prevalence of low muscle mass of 55.6% and sarcopenic obesity of 34.8%. Thus, the prevalence of low muscle mass and sarcopenic obesity was higher in older adults and people with normal BMI.

The study by Minohara et al. [45] moved from the consideration that several genetic loci related to lean mass were identified in healthy individuals; however, the contribution of these loci to body composition in T2DM remained to be investigated, which was the aim of the study. In total, 176 Japanese patients with T2DM across a wide age range (38–92 years, average of 67.4) were studied (106 men and 70 women). BIA was carried out to measure the total and segmental body composition using a commercial device (InBody770, Inbody Japan, Tokyo, Japan). The total lean mass was defined as the sum of the soft lean mass, except for lipids and bone minerals in the whole body, and appendicular lean mass was estimated as the sum of the soft lean mass of both arms and both legs. Body fat mass was defined as the sum of the lipids in the whole body. Body resistance was used to estimate the skeletal muscle mass in the whole body, according to an appropriate formula (the same formula used in the study by Rizzo et al. [28]). Relevant single-nucleotide polymorphisms were evaluated by genotyping and their contributions to body composition were examined considering known clinical determinants. Thus, one single-nucleotide polymorphism (IRS1 rs2934656) was identified as an independent predictor of skeletal muscle mass, and another one (ADAMTSL3 rs4842924) was an independent predictor of body fat mass and appendicular lean mass. It was concluded that the study findings clarified the contribution of genetic factors (IRS1 and ADAMTSL3) to interindividual variation in body composition in T2DM (independently from clinical factors), and such results can contribute to the establishment of effective methods for the prediction, prevention, and intervention for sarcopenia and frailty in diabetic patients.

In the study by Jiang et al. [46], the aim was to investigate the association between muscle mass and function (of relevance for possible sarcopenia) and the use of different glucose-lowering drugs. Data of 1042 hospitalized patients (631 males, 411 females) with T2DM were included in this study. Skeletal muscle mass (SMM) was tested with multifrequency BIA (InBody 770, Seoul, Korea) and the skeletal muscle index was equal to the SMM divided by height squared × 100. Muscle strength was tested using the handgrip strength measurement, and taking the maximum reading of at least two trials using both hands in maximum-effort isometric contraction. Physical performance was assessed using six-meter gait speed. Sarcopenia was diagnosed according to the 2019 criteria of the Asian Working Group for Sarcopenia [18]. In such criteria, low muscle mass was defined as in the original set of criteria (briefly reported above, when illustrating the study by Murai et al. [33]), whereas one update was related to low muscle strength in men, defined as handgrip strength < 28 kg (whereas it remained as before for women). In addition, in the new criteria, low physical performance was defined as six-meter gait speed < 1.0 m/s. Sarcopenia was again defined as low muscle mass and either low muscle strength or low physical performance. It was found that in patients aged ≥ 60 years, 141 out of 491 were sarcopenic. Results also showed that the skeletal muscle index, handgrip strength, and gait speed decreased in patients using acarbose compared with the other patients, using different treatments, such as metformin, sulfonylureas, DPP-4 inhibitors, or insulin. Thus, it was concluded that acarbose treatment seems to contribute to decreased muscle mass and strength; hence the assessment of muscles condition, as well as proper exercising, may be extremely relevant in patients with long-term acarbose treatment.

The very recent (2022) study by Kis et al. [47] moved from the authors’ consideration that using low handgrip strength cut-off points for the initial identification of sarcopenia, according to the 2019 guidelines of the European Working Group on Sarcopenia in Older People (EWGSOP2) [16], may mask the presence of sarcopenia. Thus, the relative knee extension strength test may help clinicians reduce false-negative results in sarcopenia diagnosis. A cohort of 100 T2DM elderly patients was studied (60% women, average age 74.5 years, mostly obese). Body composition measurements were obtained through segmental multifrequency BIA (InBody 770, InBody Co., Ltd., Seoul, Korea), by the same technician throughout the study. Measurements included fat mass, % body fat, total and segmental skeletal muscle mass (both legs, trunk, and both arms), and yielding appendicular skeletal mass index (ASMI, as the sum of skeletal masses of both arms and legs divided by height squared). In addition, patients underwent handgrip strength (HGS) and knee extension strength (KES) tests. Regression analyses were conducted to examine which variables can best predict ASMI, KES, and HGS. Results showed that using appropriate cut-off points for low KES helped to identify 24 patients with probable sarcopenia and 2 patients with confirmed sarcopenia. Conversely, using the EWGSOP2 cut-off points for low HGS, only one patient with probable sarcopenia was identified and none of the patients with confirmed sarcopenia were identified. Thus, it was concluded that KES cut-off points, attainable with a simple hand-held dynamometer, can assist in the identification of probable and confirmed sarcopenia, as identified by EWGSOP2 cut-off values for low muscle mass, especially in patients with a high BMI.

## 4. Adding Explicit Nutritional Information

In this section, we review the studies in T2DM populations where some explicit information of nutritional type or interest has been reported, in addition to the BIA-derived information of direct relevance for sarcopenia.

In 2014, the study by Akpinar et al. [48] assessed muscle mass and strength in T2DM patients, both elderly and younger, as well as in elderly and younger non-diabetic subjects. The specific aim was defining correlates of muscle mass and strength in these subjects. Sixteen elderly T2DM, sixteen younger T2DM, sixteen elderly non-diabetic, and eighteen younger non-diabetic subjects were studied. Fat-free mass (FFM) was measured by BIA using the BC-532 body analysis monitor for personal use (no further details reported). FFM was then corrected relative to height squared (CFFM). CFFM values with 2 standard derivations below the mean value of the younger non-diabetic group were defined as indicators of sarcopenic muscle mass. To assess muscle strength, isokinetic leg extension and flexion tests were performed using a dynamometer. Furthermore, during the exercise tests, in addition to functional capacity and maximum heart rate, the metabolic equivalent (MET) was recorded, which is of direct nutritional interest (though the methodology for MET measurement was not indicated). Results showed that muscle mass was similar between all groups, whereas muscle strength was significantly lower in diabetic and non-diabetic elderly subjects compared with younger diabetic and non-diabetic subjects. Muscle strength was correlated positively with MET, albumin, and hemoglobin, whereas it correlated inversely with age, HbA1c, functional capacity, and CRP. There were no clinically significant correlates of muscle mass. Of note, in this study, the presence or duration of diabetes were not found associated with muscle mass or strength. MET was found higher in younger T2DM compared to elderly T2DM subjects. The main study conclusions were that exercise test parameters may be useful markers in screening for sarcopenia; however, uncomplicated diabetes does not seem to accelerate aging-related muscle mass or strength loss.

In 2015, the study by Hamasaki et al. [49] moved from the consideration that loss of muscle mass (sarcopenia) increases the incidence of obesity by reducing physical activity; on the other, hand sarcopenic obesity may become self-perpetuating, since obesity-related reduced physical activity, increasing the risk of sarcopenia worsening. Thus, the study investigated the associations of sarcopenic indices with metabolic parameters related to obesity, in patients with T2DM. Selected sarcopenic indices were the ratio of lower extremity muscle mass to body weight (L/W ratio), and the ratio of lower extremity muscle mass to upper extremity muscle mass (L/U ratio). A group of 26 T2DM patients with obesity (BMI over 30.0 kg/m^2^) but no physical disability was studied (10 men and 16 women, aged 27 to 76 years old). Body composition was analyzed using BIA (InBody720; Biospace Co., Ltd., Tokyo, Japan). In more detail, segmental body composition was estimated using a patented eight-point tactile electrode system. Similar to what reported in a study illustrated above [38], the device used 6 frequencies (1, 5, 50, 250, 500, and 1000 kHz) and produced 30 impedance values for five body segments, i.e., right and left upper extremities, trunk, and right and left lower extremities. Of note, it was clarified that previous validation studies showed that both fat mass and lean mass, evaluated by the indicated methodology, were highly correlated with those measured by dual-energy X-ray absorptiometry (with a correlation coefficient of 0.832 and 0.899, respectively). Furthermore, in this study, visceral and subcutaneous fat areas were measured using abdominal computed tomography. In addition, daily physical activity was measured by a triaxial accelerometer during a period of hospitalization. The used device differentiated 11 daily activities with almost 100% accuracy, and quantified the metabolic equivalent values (METs), which were strongly correlated with METs calculated from energy expenditure, as measured by indirect calorimetry. For METs, other parameters (such as the total energy expenditure) were derived. It was found that the L/W ratio was negatively correlated with BMI, body fat mass, subcutaneous fat area, and other parameters (such as serum free fatty acid concentration), whereas it was positively correlated with daily physical activity. The L/U ratio was positively correlated with serum HDL cholesterol. It can be concluded that high L/W and L/U ratios, indicative of preserved lower extremity muscle mass, were predictive of improved metabolic parameters related to obesity. Thus, in obese people with T2DM, preserved muscle fitness (especially of the lower extremities) may prevent sarcopenic obesity.

In 2019, the study by Küçükdiler et al. [50] aimed to evaluate the relationship between sarcopenia and oxidative stress, as well as antioxidant status, in elderly patients with T2DM. In fact, it was noted that oxidative stress may play a role in the pathogenesis of both sarcopenia and T2DM; however, the relationship between sarcopenia oxidative stress and antioxidant status among the older T2DM population was not well investigated at the time of the study. In total, 60 T2DM elderly patients (≥65 years, BMI < 30 kg/m^2^) were enrolled (30 sarcopenic and 30 controls, with 19 females and 11 males in each group). Sarcopenia was assessed according to the EWGSOP criteria [15,16], which consider gait speed and handgrip strength, in addition to skeletal muscle mass. The latter was assessed by BIA, which was performed using the Quadscan 4000 body composition analyzer (Bodystat, P.O. Box 50, Douglas, Isle of Man, IM99 IDQ, British Isles). A resistance value at 50 kHz was also considered. The equation that was used to calculate skeletal muscle mass is the same already seen in some of the studied illustrated above, as in the study by Rizzo et al. [28]. After calculating the skeletal muscle mass, the absolute skeletal muscle mass was obtained by normalizing to height squared. Values less than 8.87 kg/m^2^ and 6.42 kg/m^2^ indicated low skeletal mass for men and for women, respectively. As regards the other sarcopenia-related parameters, a 4 m gait speed test was performed, with participants walking at their usual speed; in the case of speed ≤ 0.8 m/s, a low speed was classified. Handgrip strength was measured with a handheld digital dynamometer; two measurements were taken from the dominant hand and the highest value was recorded for each patient; and low handgrip strength was defined as <30 kg for men and <20 kg for women. Furthermore, within a comprehensive geriatric assessment, information on nutritional status were collected using an appropriate survey (the so-called “mini nutritional assessment short form”). In addition, several parameters reflecting oxidative stress and antioxidant status were measured, such as the plasma and the erythrocyte malondialdehyde, the glutathione peroxidase, the superoxide dismutase, the catalase, and xanthine oxidase. Results showed that plasma xanthine oxidase was independently associated with sarcopenia; thus, it can be important in the pathogenesis of sarcopenia in diabetes. The score derived by the mini nutritional survey was, however, not different between sarcopenic and control subjects. It was concluded that oxidative stress and antioxidant status might be associated with sarcopenia in older T2DM individuals; however, this association seems to be mediated by other factors.

In 2019, the study by Okamura et al. [51] investigated the relationship between sarcopenia and energy intake in T2DM elderly patients, which is an important factor for the maintenance of muscle mass. In total, 391 physically active T2DM patients aged ≥ 65 years (205 men, 186 women) were studied. The body composition was evaluated using the InBody 720 BIA device (InBody Japan, Tokyo, Japan). Body fat mass, skeletal muscle mass, and appendicular muscle mass were obtained, and the skeletal muscle mass index (SMI) was then calculated by dividing the appendicular muscle mass by height squared. Sarcopenia was diagnosed with SMI and grip strength based on the criteria from the Asian Working Group for Sarcopenia [17]. In addition, a brief-type diet history questionnaire (BDHQ) was administered to assess patient’s habitual food and nutrient intake. BDHQ helped to estimate dietary intake for 58 items over the past month, especially the daily intake and type of rice and miso soup, the frequency of consumption of several food and both alcoholic and non-alcoholic beverage items, the usual cooking methods, and general dietary behavior information. Energy, carbohydrate, protein, and fat intake were calculated using the ad hoc computer algorithm, exploiting Japanese standard tables of food composition. Thus, dietary total energy (kcal/day) and carbohydrate, protein, fat, and alcohol intake (all in g/day) were estimated using these calculations. The patients who reported extremely low (under 600 kcal) or high (over 4000 kcal) energy intake were excluded. It was found that 55 patients (14.1%) had sarcopenia. Energy intake was significantly lower in patients with sarcopenia than without sarcopenia, and after adjusting for age, sex, exercise, smoking status, HbA1c, and BMI, the energy intake was negatively associated with the presence of sarcopenia. It was concluded that low energy intake is a risk factor for sarcopenia in T2DM elderly patients.

In the same year, Okamura et al. [52] also investigated the association between sarcopenia and brain natriuretic peptide (BNP) in T2DM patients. In fact, it was noted that association between heart failure and sarcopenia had been previously reported; however, the possible association between sarcopenia and BNP, which is a biomarker of heart failure, was unclear. In total, 433 T2DM patients were studied (236 men, 197 women, average age of 65.4 years, BMI < 30 kg/m^2^). Similar to the previous study [51], body composition was evaluated using the InBody 720 device (InBody Japan, Tokyo, Japan). BIA-measured skeletal muscle mass, appendicular muscle mass, body fat mass, and the skeletal muscle mass index (SMI) were calculated as appendicular muscle mass normalized to height squared. Sarcopenia was diagnosed with the SMI and grip strength according to the Asian Working Group for Sarcopenia criteria [17], similar to the previous study [51]. Again, a brief-type self-administered diet history questionnaire was used to assess the habitual food and nutrient intake, assessing the dietary total energy (kcal/day), as well as the carbohydrate, protein, fat, and alcohol intake (g/day). Results showed that 32 out of 433 patients (7.4%) had sarcopenia. BNP levels were associated with the presence of sarcopenia, and the optimal cut-off point for BNP levels, indicating risk for sarcopenia, was determined as 27.3 pg/mL. It was concluded that in T2DM non-obese patients without heart failure, BNP levels are associated with sarcopenia.

In 2020, the study by de Freitas et al. [53] aimed to establish the prevalence of sarcopenia and associated factors in elderly patients with T2DM, according to both 2010 and 2019 criteria of the European Working Group on Sarcopenia in Older People (EWGSOP [15] and EWGSOP2 [16], respectively). In total, 242 T2DM patients ≥ 60 years were studied (54% women, 78% white, average BMI of 29.5 kg/m^2^). Muscle mass was assessed by BIA (InBody 230, no further details reported), which provided total and segmental muscle mass through the arms and legs. These values were used to calculate appendicular skeletal muscle mass (ASM) as the sum of the muscle mass of arms and legs. The skeletal muscle mass index (SMI) was determined by ASM divided by height squared. Muscle strength was assessed using the handgrip strength test, and physical performance was assessed using the timed-up-and-go test. For the latter, EWGSOP and EWGSOP2 cut-off values were the same (impaired if higher than 20 s), whereas there were differences for the SMI and the handgrip test. Indeed, according to EWGSOP, a low SMI was below 8.5 kg/m^2^ in men and 5.75 kg/m^2^ in women; however, for EWGSOP2, it was below 7.0 and 6.0 kg/m^2^, respectively. As regards handgrip strength, for EWGSOP, low values were below 30 kg and 20 kg in men and women, respectively; however, for EWGSOP2, low values were below 27 and 16 kg, respectively. As regards nutritional information, a usual diet was assessed through a food frequency questionnaire specifically validated in T2DM. The reported intake of food groups was converted into daily consumption (also expressed as a percentage of the total energy), and a Brazilian food composition table was used to evaluate the nutritional composition of the questionnaire items. It was found that the overall prevalence of sarcopenia was 21% (i.e., with either EWGSOP or EWGSOP2 criteria). In more detail, EWGSOP sarcopenia prevalence was 17%, whereas it was 7% with EWGSOP2, suggesting 3% prevalence with both criteria. In addition, age and male sex were found to increase the prevalence of sarcopenia, whereas walking (>5400 steps/day) had a strong protective effect. In contrast, nutritional information did not appear to provide relevant information regarding sarcopenia, with only carbohydrate intake being higher in patients with sarcopenia.

In a 2020 study, Okamura et al. [54] investigated the relationship between sarcopenia and omega-3 fatty acid intake in elderly T2DM patients, which is known to be important to maintain muscle mass. In total, 342 patients (180 men, 162 women) aged >65 years, with no evidence of physical inactivity, were studied. Body composition was determined by BIA (InBody 720, InBody Japan, Tokyo, Japan)to assess body fat mass, skeletal muscle mass, and appendicular muscle mass, and to calculate the skeletal muscle mass index, as carried out in previous studies by Okamura et al. [51,52], illustrated above. Diagnosis of sarcopenia was based on the Japan Society of Hepatology guidelines, similar to the study by Osaka et al. [31], as previously illustrated. For the assessment of habitual food and nutrient intake, the brief-type self-administered diet history questionnaire was used, similar to what described in some of the previous studies [51,52]. Results showed sarcopenia prevalence at 13.2% (45 patients). Patients with sarcopenia had a higher age and lower BMI than those without sarcopenia. In addition, omega-3 fatty acid intake in patients with sarcopenia was lower; on the other hand, omega-3 fatty acid intake was negatively associated with sarcopenia presence after adjusting for age, sex, exercise, smoking status, diabetes duration, and HbA1c, as well as energy, protein, and fat intake. Thus, it was concluded that omega-3 fatty acids increase muscle mass and improve skeletal muscle strength, likely by promoting neuromuscular function.

In 2021, the study by Oguz et al. [55] aimed to evaluate sarcopenia and sarcopenic obesity in patients with T2DM, and the possible relationships of sarcopenia with serum irisin and myostatin levels. A group of 90 T2DM patients (20 males, 70 females), aged 18–70 years, with a BMI of 25–40 kg/m^2^, was studied. Body composition was measured by BIA (TANITA DC 360 ST, Tokyo, Japan). In the article’s methods, it is specified that patients were asked to not eat, drink, or undertake any physical activity at least three hours before the test, and to void the bladder immediately before the measurement. Fat mass, fat-free mass, and appendicular skeletal muscle (ASM) measurements were recorded. The skeletal muscle index (SMI) was calculated as ASM divided by height squared, and %ASM as ASM divided by body weight × 100. Handgrip strength tests and 6 m gait speed tests were also used; hence, sarcopenia was diagnosed according to the 2019 EWGSOP criteria [16]. Myostatin and irisin levels were measured using commercially available solid-phase sandwich enzyme-linked immunosorbent assay (ELISA) kits. As regards specific dietary information, it was stated that dietary compliance in the self-management of diabetes was recorded (though details were not provided). It was found that prevalence of sarcopenia and sarcopenic obesity was 25.6% and 35.6%, respectively. Irisin levels were lower in sarcopenic patients. Irisin remained an important predictor of sarcopenic obesity, even after adjusting for confounding variables, with an optimal cut-off value for sarcopenic obesity prediction by irisin equal to 9.49 ng/mL (specificity = 78.1%, sensitivity = 75.8%). Moreover, HbA1c was an independent risk factor for sarcopenic obesity. Conversely, the rate of dietary compliance was not different between sarcopenic and non-sarcopenic patients (about 48%). It was concluded that low irisin levels and poor glycemic control in T2DM patients are independent risk factors for sarcopenia, especially for sarcopenic obesity.

## 5. Longitudinal and Interventional Studies

In this section, we review the articles related to investigations that included a follow-up period of the studied subjects, possibly with intervention, such as pharmacological treatment.

In 2018, the study by Sugiyama et al. [56] aimed to investigate the effects on muscle mass and fat content of sodium–glucose co-transporter 2 inhibitor (SGLT2i) treatment (dapagliflozin). A group of 50 Japanese T2DM patients (HbA1c > 7%) was prospectively recruited, with BMI < 35 kg/m^2^ (72% males, average age of 56.1 years). Patients were divided into two groups: one treated with dapagliflozin (5 mg/day) and the other one with non-SGLT2i agents. Treatment lasted six months, with HbA1c improvement as primary outcome. Body composition including total fat mass, soft lean mass, and skeletal muscle mass was measured using a segmental multifrequency BIA device (InBody 770). Similar to what reported in studies illustrated above [38,49], the device used tetrapolar eight-point tactile electrodes and processed 30 impedance measurements at a different frequencies (1, 5, 50, 250, 500, and 1000 kHz) on five body segments (right arm, left arm, trunk, right leg, left leg), as well as 15 reactance measurements at three different frequencies (5, 50, and 250 kHz) on the same five body segments. The skeletal muscle mass index, calculated as quantitative indicator of sarcopenia, was computed as skeletal muscle mass normalized to height squared. In addition, the psoas muscle area (other indicator of total skeletal muscle mass) was assessed using abdominal computed tomography, and the psoas muscle index was computed by normalization again for height squared. After the follow-up period, it was found that HbA1c, body weight, and BMI were significantly decreased in both treatment groups, and the HbA1c decrease was not significantly different between groups. In addition, dapagliflozin treatment decreased total fat mass, without affecting the skeletal muscle mass index. In fact, the absolute change in soft lean mass and skeletal muscle mass was not different between groups. Moreover, dapagliflozin treatment did not decrease the psoas muscle index, and its absolute change was not different between groups. It was concluded that in T2DM patients, treatment with dapagliflozin for six months significantly improved glycemic control and reduced body weight without reducing muscle mass. Thus, dapagliflozin appears a proper agent in terms of the effects on the balance between fat and muscle mass.

In 2021, the study by LeCroy et al. [57] aimed to determine whether loss of muscle mass is associated with risk for T2DM in Hispanic/Latino adults. Study participants were 6264 Hispanic/Latino adults (48.4% males, 18–74 aged years) without T2DM at baseline, which were prospectively followed on average for 6.1 years. At baseline and at the follow-up visit, participants underwent BIA (Tanita Body Composition Analyzer, TBF-300A; Tanita Corporation, Tokyo, Japan) to measure fat mass and fat-free mass (FFM), which was assumed as a marker of muscle mass. The relative FFM was then defined as FFM divided by body weight, and a percentage change in the relative FFM (%ΔFFM) was calculated as the difference between relative FFM_baseline_ and relative FFM_follow-up_ divided by relative FFM_baseline_. In addition, sarcopenia was defined as having FFM ≥ 2 standard deviations below the sex-specific average FFM of healthy young (20–29-years old) Mexican Americans. Results showed that relative FFM declined by 2.1% between visits, and that %ΔFFM was inversely associated with changes in glucose and insulin levels, and with incident prediabetes. Associations were generally stronger for individuals with baseline overweight or obesity. The prevalence of sarcopenia was very low (less than 1%); however, in our opinion, this may be due to the assumed criterion for sarcopenia identification, which does not agree with those of the official recommendations. It was concluded that reducing loss of FFM during adulthood may reduce prediabetes risk (primarily insulin resistance), particularly among individuals with overweight/obesity.

The 2021 study by Low et al. [58], examined among Asian T2DM patients, showed longitudinal relationships between changes in skeletal muscle mass and those in the estimated glomerular filtration rate (eGFR) and in albuminuria, since in previous cross-sectional analyses it was shown that sarcopenia is associated with lower eGFR. In total, 1272 T2DM patients were prospectively studied (616 men, 656 women, average age of 58.8 years, 693 subjects in in the 40–59 years range). Body composition was measured using tetrapolar multifrequency BIA at baseline (InBody-S20; Biospace, Cerritos, CA, USA) and at one follow-up visit (InBody-S10; Biospace, Cerritos, CA, USA) over a median period of 3.2 years, with a maximum of 7.8 years. The skeletal muscle mass index (SMI) was defined as total skeletal muscle mass/weight × 100. It was found that, at follow-up, about one third of participants had progression of chronic kidney disease (CKD), as shown by proper criteria based on eGFR decline, as well as albuminuria progression. The largest decrease over time from baseline SMI (first tertile) was associated with more than 60% higher risk of kidney disease progression, compared to that of the lowest tertile. Accordingly, every improvement in one standard deviation above baseline SMI was associated with about 20% lower risk of kidney disease progression and albuminuria progression. The main conclusion, therefore, was that low baseline skeletal muscle mass and its reduction over time is associated with increased risk for progression of CKD in Asian T2DM patients. 

A retrospective longitudinal study was carried out by Buscemi et al. [59], with focus on women. The main aim was to investigate whether body composition analysis predicts the development of impaired fasting glucose (IFG) or T2DM in a cohort of elderly women (≥65 years, average age of 71 years). In total, 159 women were included (with normal glucose tolerance at baseline) over a follow-up period of 94 months. Hand-to-foot BIA was performed (BIA-EFG, Akern srl, Florence, Italy) to estimate the body resistance, reactance, phase angle, percentage of fat mass (FM), and appendicular skeletal muscle mass (ASMM), according to the manufacturer’s equations (Akern, Bodygram Plus software). In agreement to EWGSOP2 guidelines [16], a cut-off value of 15 kg was used for diagnosing BIA-derived low ASMM. FM was exploited to define obesity (FM ≥ 35%). Sarcopenia was defined again in agreement with EWGSOP2 criteria, thus considering the presence of low muscle strength (handgrip strength < 16 kg) in addition to low ASMM. Furthermore, in this study, some explicit nutritional information was also collected. In fact, dietary intake was assessed via combination of a validated food frequency questionnaire and a 7-day food record, and it was calculated using proper nutritional software (MetaDieta 3.0.1, Metedasrl, San Benedetto del Tronto, Italy). The database used to calculate the nutrient intake was primarily derived from the National Institute of Food Research (INRAN) 2000 and the European Institute of Oncology (IEO) 2008. Results showed that participants with low ASMM had a higher IFG/T2DM incidence than those with normal ASMM over time, independently from BMI, fat mass, and habitual fat intake. The prevalence of sarcopenia at baseline was 9% and that of low ASMM was 45%. In the low ASMM subgroup, higher body resistance and lower body phase angle were also detected. Higher incidence of IFG/T2DM was observed in the subgroup with sarcopenia compared to those without sarcopenia, independently from BMI and fat mass. Thus, the study demonstrated that elderly women with low ASMM or sarcopenia had a higher probability of developing IFG/T2DM. In our opinion, this is in line with the hypothesized bidirectional relationship between sarcopenia and T2DM. Thus, not only is T2DM a risk factor for sarcopenia, but also sarcopenia (deteriorated muscle mass) is a risk factor for T2DM.

The study by Lee et al. [60] aimed to examine the long-term longitudinal association between the loss of muscle mass and the presence of T2DM or CKD. This study had somehow similar aims compared to the study by Low et al. [58]; however, the recruited population was larger and the follow-up period was longer. Indeed, 6247 middle-aged adults (48.1% males, average age of 51.2 years) were studied, for a period up to 15 years (between 2001 and 2016). Patients were classified into four groups according to the presence or absence of T2DM and CKD. Body composition was assessed using multifrequency BIA (InBody 3.0, Biospace, Seoul, Korea), which was performed at baseline and biennially during the entire study period. Of note, it was explicitly clarified that multifrequency BIA has advantages compared to conventional BIA, since the latter relies on formulae to calculate the estimated mass of each body component, whereas the former assumes that the human body consists of five interconnecting cylinders and performs impedance measurements directly on these compartments (i.e., arms, trunk, and legs). Impedances were measured at four specific frequencies (5, 50, 250, and 500 kHz) using a tetrapolar eight-point tactile electrode system. The primary endpoint was de novo development of muscle depletion, which was defined as a decline in the BIA-derived fat-free mass index (FFMI) below the 10th percentile of the total population sample (for males < 16.9 kg/m^2^, and for females < 15.2 kg/m^2^). Secondary outcomes included the occurrence of cachexia (well known to have relationships with sarcopenia) and all-cause mortality. During 73,059 person-years of follow-up (median follow-up of 13.7 years), muscle depletion occurred in about 7% of the subjects. The risk of muscle depletion was higher in subjects with T2DM than in those without T2DM and CKD, and it was strongly pronounced in subjects with both T2DM and CKD. Accordingly, the annual decline rates in FFMI (but also in BMI) were the steepest in subjects with both T2DM and CKD. The secondary outcome analyses showed consistent results. It was concluded that T2DM and CKD are synergically associated with muscle loss over time, and the mortality risk is higher in individuals with muscle loss.

The study by Sundar et al. [61] aimed to identify the association of sarcopenia and T2DM with clinical outcomes among hospitalized cardiac patients. This prospective observational study assessed 100 patients (76 males, 24 females, average age of 60.0 years, 82% overweight/obese, 50% with T2DM), which were followed-up until hospital discharge 90 days thereafter. Multifrequency BIA (Seca mVSA 531, Hamburg, Germany) was used to measure body composition, including skeletal muscle mass and fat mass. Patients were asked to void their bladder before the measurement, which was performed 10 min after the patients presumed in supine position on the bed, with arms and legs apart from the trunk. The appendicular skeletal muscle mass index (ASMI) was calculated as appendicular skeletal muscle mass divided by height squared. The handgrip strength test was also performed, and sarcopenia was diagnosed in both low ASMI and low handgrip strength cases, with cut-offs established by the guidelines of the 2019 Asian Working Group for Sarcopenia [18]. Prognosis of the patients was assessed using the prognostic nutritional index (PNI) calculated as 10 × serum albumin (g/dL) + 0.005 × lymphocyte count (per mm^3^). The outcome measures were the length of hospital stay (LOS), unplanned readmission within 90 days after discharge, and incidence of infections within 90 days after discharge. It was found that the prevalence of sarcopenia was 63%, and this was similar in patients with or without T2DM. After adjustments, sarcopenia was associated with 90-day unplanned readmission and LOS, whereas the condition of co-existence of sarcopenia and T2DM was associated with 90-day unplanned readmission and 90-day incidence of infections. It was concluded that sarcopenia with co-existent T2DM is associated with increased risk for hospital readmission and infections among cardiac patients. Thus, early identification of sarcopenia may be important for timely intervention to improve prognosis in cardiac T2DM patients.

Koo et al. [62] aimed to assessed the determinants of glycemic control in elderly people with T2DM, including pancreatic beta-cell function, insulin resistance, muscle mass, and muscle quality. This was a prospective study in T2DM patients aged ≥ 60 years with a T2DM duration of ≥10 years. In total, 100 patients were studied (49% men), whose baseline average characteristics were age of 64 years, BMI of 24 kg/m^2^, and HbA1c of 7.1%. The median follow-up duration was 4.0 years. BIA was performed using the InBody 330 analyzer (InBody, Seoul, Korea). Muscle mass was expressed as a percentage (muscle/weight × 100) and similarly for fat mass, and their quartiles were calculated for each sex. The handgrip strength test was also performed, and muscle strength values are again stratified in sex-related quartiles. Low muscle mass and low muscle strength were defined according to the EWGSOP2 criteria [16]. Beta-cell function and insulin resistance were calculated from a 75 g oral glucose tolerance test (OGTT). Results showed that low muscle strength and insulin resistance at the baseline were independent determinants of the primary study outcome, i.e., HbA1c deterioration, following adjustment for age, sex, obesity, T2DM duration, antidiabetic medication use, and baseline HbA1c. In addition, sex stratification showed that, in women, both muscle strength and muscle mass were independent determinants of the primary outcome. The main conclusion was that, overall, low muscle strength and insulin resistance are the main risk factors for aggravated glycemic control among elderly patients with long-standing T2DM.

The study by Hasegawa et al. [63] aimed to investigate the effect of COVID-19 pandemic restrictions on changes in muscle mass in older patients with T2DM. Data were obtained from patients who underwent BIA at least twice before April 2020 and at least once thereafter. Thus, 56 patients aged > 60 years were recruited (35 men and 21 women, average age of 75.2 years). Body composition was evaluated using a multifrequency impedance BIA (InBody 720, InBody Japan, Tokyo, Japan). Data on appendicular muscle mass and fat mass were collected. The skeletal muscle mass index (SMI) was determined using the appendicular muscle mass divided by height squared. Percent fat mass was calculated as fat mass divided by body weight × 100. Changes in the SMI (kg/m^2^/year) were calculated as (follow-up SMI—baseline SMI)/follow-up period (in years). Similarly, changes in body weight, appendicular muscle mass, and body fat and percent body fat were also calculated. In addition, low muscle mass was defined as SMI < 7.0 kg/m^2^ in men and <5.7 kg/m^2^ in women. Obesity was considered for BMI ≥ 25 kg/m^2^. Results showed a slight improvement in the SMI before the COVID-19 pandemic, whereas the SMI significantly decreased after the start of the COVID-19 pandemic. This decrease was particularly evident in men, in patients with poor glycemic control, and in those with a long diabetes duration. It was concluded that COVID-19 pandemic restrictions caused muscle mass loss in older patents with T2DM; thus, recommendations for exercise and adequate diet intake should be provided in such a population to prevent loss of muscle mass and, hence, risk for sarcopenia.

The very recent (2022) study by Low et al. [64] aimed to study the profile of amino acids longitudinally associated with skeletal muscle mass loss in T2DM. This was a prospective study in 1140 T2DM patients (591 males, 549 females, average age of 56.6 years), followed for a period up to 7.9 years. Skeletal muscle mass was measured using tetrapolar multifrequency bioimpedance analysis at baseline (InBody-S20; Biospace, Cerritos, CA, USA) and at follow-up (Inbody-S10; Biospace, Cerritos, CA, USA). The skeletal muscle mass index (SMI) was defined as total skeletal muscle mass/weight × 100. Cut-off values for a low SMI were assumed as 30.8% in men and 24.3% in women, according to the guidelines from the 2019 Asian Working Group for Sarcopenia [18]. Changes in SMI were calculated as the follow-up SMI minus the baseline SMI. Amino acids were measured by mass spectrometry. Results showed that 43.9% subjects experienced skeletal muscle mass loss. Lower baseline valine, leucine, and isoleucine levels were associated with decreased SMI. Therefore, the main conclusion was that, in T2DM, branched-chain amino acids (valine, leucine and isoleucine) appear to have a preventive role in muscle mass loss over time, though it was acknowledged that further studies should be conducted to elucidate the pathophysiological mechanisms underlying the relationship between these amino acids and muscle mass loss.

Another very recent study was carried out by Hoppe et al. [65], which aimed to investigate the impact of T2DM on selected indicators of protein–energy wasting in end-stage renal disease (ESRD) patients, undergoing maintenance hemodialysis (MHD). The study moved from the notion that ESRD is a deteriorating catabolic condition predisposing patients to protein–energy wasting, which is in contrast to T2DM, i.e., a systemic metabolic disease typically associated with overnutrition and consequent overweight/obesity. Based on this, the study intended to investigate the potential paradox of T2DM as a risk factor of protein–energy wasting development in ESRD patients under MHD. The multicenter, prospective, observational study was performed in a cohort of 515 ESRD patients (310 males and 205 females, median age of 67.3 years), followed for a period up to 5 years. Patients were divided into two subgroups, i.e., with and without T2DM (198 and 317, respectively). BIA was performed with a body composition monitor (Fresenius Medical Care Deutschland GmbH, Bad Homburg, Germany) to determine body composition parameters and hydration status. Precisely, the lean tissue mass and fat tissue mass, lean tissue index and fat tissue index (normalization to height squared), overhydration, and relative overhydration were measured. BIA was performed in supine position using disposable electrodes, which were attached to the hand and foot, contralateral to the arteriovenous fistula. Both BIA and the other study measurements were performed shortly before a mid-week hemodialysis session. Specific nutritional information was also detected. In fact, the quantitative evaluation of diet composition was performed in terms of the intake of energy and different nutritional components (proteins, lipids, cholesterol, carbohydrates, fiber, sodium, and potassium). Total metabolic rate, as an indicator of adequate nutritional requirements, was calculated based on body weight, height, age, sex, and activity level. Patients prepared a three-day food diary, including one hemodialysis day and two non-dialysis days. Food intake expressed in grams was converted to nutrient intake using a dietary calculator based on data from the National Nutrient Database for Standard Reference by the United States Department of Agriculture (USDA). In addition, a dialysis-modified questionnaire estimated the subjective global assessment (SGA) score, which is used to assess the protein–energy wasting on the basis of the patient’s history of nutrition quantity and quality, combined with anthropometric measurements of fat and muscle mass variations. SGA scores ranged from 7 (proper nutritional state) to 35 (severe malnutrition). Results showed that, compared to non-diabetic patients, T2DM patients had a higher SGA score, BMI, fat tissue mass and index, and overhydration/relative overhydration, whereas they had lower albumin, total cholesterol, and creatinine. Furthermore, increased morbidity and mortality was also observed in T2DM patients, for both cardiovascular diseases and other causes. It was concluded that hemodialysis patients with T2DM, on the one hand, show overnutrition, but a paradoxically higher predisposition to protein–energy wasting, expressed by a higher SGA score, on the other, as well as lower serum markers of nutrition. Of note, it was also suggested that higher protein–energy wasting, despite higher BMI and caloric intake, observed in T2DM, may be indicators of obese sarcopenia. However, in our opinion, it was however somehow surprising that the lean tissue mass and index were not different between T2DM and non-T2DM.

## 6. Concluding Comments

In this review study, we summarized the studies in patients with T2DM and sarcopenia (or at risk for it), where BIA is used for body composition analysis. For easier readability, we grouped the revised studies into three main sections, i.e., one for the cross-sectional studies, one for cross-sectional studies also including specific nutritional information (that we have emphasized in our analysis), and one for the longitudinal studies, either observational or interventional.

First, it is worth recalling that BIA is important for the diagnosis of sarcopenia, since it is among the techniques for the assessment of muscle mass (this being one of the sarcopenia factors) that are deemed acceptable by different international guidelines [15,16,17,18,19]. Of note, in the studies examined in this review, possible differences of sarcopenia prevalence in T2DM, according to different guidelines, has also been addressed. However, apart for sarcopenia diagnosis, in the context of T2DM-related sarcopenia, BIA appears useful for several purposes. In fact, our review shows that, in T2DM patients with sarcopenia (or at sarcopenia risk), BIA has proven relevant for several scientific and clinical goals, as it has been exploited in studies with very different outcomes. Some studied have contributed to the knowledge of the muscle mass condition in T2DM compared to healthy (or at least non-T2DM) control subjects, with analyses of the muscle mass either at whole body level or at specific body segments (especially lower limbs). Other studies have shown the associations between muscle mass deterioration (and possibly overt sarcopenia) in T2DM with different diabetes-related complications, such as atherosclerosis and microcirculation impairment, chronic kidney disease, hepatic steatosis and specifically non-alcoholic fatty liver disease, diabetic neuropathy, infections, and even cognitive dysfunction. Furthermore, some studies showed associations of T2DM-related sarcopenia/muscle mass loss with specific disorders or pathophysiological conditions, such as high levels of HDL-cholesterol, oxidative stress, high blood pressure variability, as well as genetic factors. In addition, abnormal values of some serum/plasma parameters were suggested as possible markers of muscle loss and possibly sarcopenia, such as the creatinine to cystatin C ratio, irisin, sclerostin, and brain natriuretic peptide. On the other hand, some studies showed the beneficial effect of nutraceuticals or dietary supplements in increasing muscle mass or at least preventing muscle mass loss, such as omega-3 fatty acids and branched-chain amino acids. Some studies also investigated the effects on muscle mass of some antidiabetic agents, such as DPP-4 inhibitors, sulphonylureas, SGLT2 inhibitors, and acarbose, showing that some agents appear beneficial, whereas others may be harmful.

**Figure 3:**
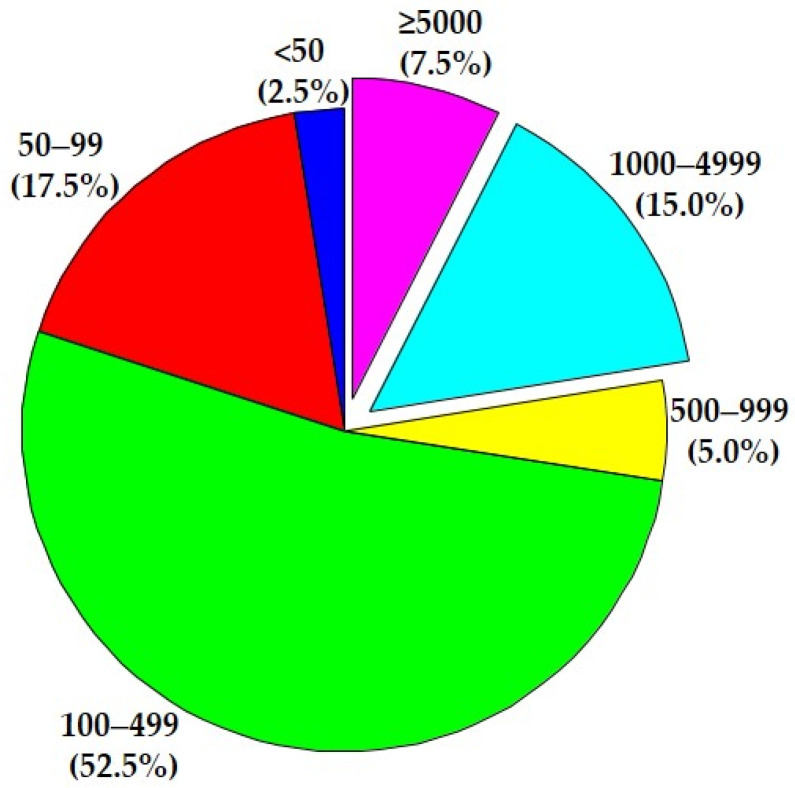
Percentage distribution of the studies analyzed in the review in terms of the size of the study cohorts (from minimum of <50 subjects to maximum of ≥5000 subjects).

One study also showed the negative effects of the COVID-19 pandemic restrictions, in terms of muscle mass loss in elderly patients with T2DM. In summary, in patients with T2DM with sarcopenia or at sarcopenia risk, BIA has been extensively used for the study of body composition (especially for muscle mass assessment). Notably, due to non-invasiveness, simple execution, and relatively low cost, BIA appears adequate for use in clinical practice, and this is mirrored by the large number of patients analyzed in the studies addressed in this review. Figure 3 shows the percentage of studies at different size of the studied cohort (<50 subjects, 50–99 subjects, 100–499 subjects, 500–999 subjects, 1000–4999 subjects, ≥5000 subjects). 

It is interesting to observe that more than 20% of the studies included more than 1000 subjects (about 8%: more than 5000). Overall, in the 40 studies addressed in this review, almost 39,000 subjects were studied. In our opinion, this is a clear indication that investigators consider BIA as trustworthy for the study of body composition (and, specifically, muscle mass) in T2DM patients with sarcopenia or at risk of the disease. The acceptance of the scientific community for such approach is further indicated by the high number of citations of some of the revised articles, in relation to the typically recent publication year, as shown in Table 1 (on the other side, absence of citations for some articles is likely due to the very recent publication date). In our opinion, this further suggests that study methodologies were deemed reliable, obviously including the BIA approach for the analysis of muscle mass, which was a key issue in all examined studies.

On the other hand, one may wonder: is current evidence in terms of BIA reliability totally satisfactory for investigations in T2DM patients with sarcopenia or at least with muscle mass loss? It has been reported that several studies demonstrated the validity of both single-frequency and multifrequency BIA, concluding that BIA may be used as an alternative to more complex/expensive techniques, such as DXA, for whole-body and segmental body composition assessment, especially in large cohorts [14]. However, it has also been documented that some studies demonstrated differences when comparing BIA to DXA, especially for segmental measures or with single frequency devices, with the inaccuracy increasing with higher BMI levels [14]. Interestingly, it was also reported that the accuracy of different BIA devices may vary, and may depend on the specific body composition parameters assessed, as well as on the equations used by the device in question, with many manufacturers using their own equations derived during the internal validity testing of the product [14,66,67]. This may result in equations that are general, with little specificity to varying populations. Based on these premises, in our opinion, it is worth noting that BIA accuracy should have been investigated in the specific population of patients with T2DM and concomitant sarcopenia. However, to our knowledge, no study focused on BIA validation in a T2DM sarcopenic population. Such kind of study may be relevant to clarify whether BIA is really sufficiently reliable (as we expect) in T2DM sarcopenic patients and, possibly, under what peculiar conditions/circumstances special caution should be required. In other words, such a study can draw the lines for “guaranteed” use of BIA in T2DM sarcopenic patients, leading to greater awareness in BIA use for such a population. 

Another question may be as follows: assuming that BIA can be deemed as reliable in T2DM sarcopenic/low muscle mass population, are there nonetheless limitations regarding the use of BIA in the studies analyzed in this review? In essence, the great majority of analyzed studies assessed the same parameters of body composition. Investigated parameters were typically fat mass and fat-free/skeletal muscle mass at a whole body level and sometimes at different body regions (e.g., arms, legs, and possibly trunk), usually normalizing to body weight or height squared (though not always motivating the preference for one or the other normalization). Just a few studies investigated other parameters that BIA can provide (at least, the more modern devices), such as intracellular, extracellular, total body water, and bone mineral content. The lack of analysis of such “advanced” body composition parameters is common to many of the analyzed studies. In addition, for improved accuracy, BIA measurements should be consistently taken in similar conditions among patients (for instance, in the morning at fasting, to limit the effect of differences in the state of hydration of the patients). However, surprisingly, the majority of the revised studies did not clarify under what specific conditions BIA was performed, thus this is another quite common limitation. On the other hand, it has to be acknowledged that the overall quality of the revised studies was acceptable in our opinion, and even remarkable in some cases. In fact, we analyzed the revised studies according to the “Quality Assessment Tool for Observational Cohort and Cross-Sectional Studies” (see https://www.nhlbi.nih.gov/health-topics/study-quality-assessment-tools; last checked: 15 April 2022). This tool suggests criteria to rate the articles and finally classify them in one of three quality categories, i.e., “poor”, “fair”, and “good”. Based on such criteria and indications, we did not rate any article as “poor”. As regards the 30 cross-sectional studies (see Section 3 and Section 4 of this review), we rated 15 of them as fair and 15 as good. For the 10 longitudinal studies (see Section 5), we rated 3 studies as fair and 7 studies as good.

In conclusion, we examined the studies where BIA is used to analyze body composition in T2DM patients with sarcopenia or at risk of it. The revised studies have shown to provide several important pieces of information concerning the relationships between body composition parameters (mainly muscle mass) and other aspects of T2DM patients’ conditions, including different comorbidities, as well as information on how to oppose to muscle mass deterioration. Such relevant findings suggest that BIA can be considered generally appropriate for body composition analysis in T2DM complicated by sarcopenia/muscle loss. In addition, the wide size of the patients’ cohort in many studies indicates that BIA is particularly adequate and convenient for clinical applications. On the other hand, future studies may pay more attention to better clarify the operating conditions assumed for BIA measurement, and a larger battery of parameters may be studied for a more complete picture of the body composition state. Furthermore, in our opinion, studies should be carried out with focus on the specific validation of BIA performances, in the peculiar population of patients with T2DM complicated by sarcopenia.

## Figures and Tables

**Table 1:** Main characteristics and information on analyzed studies. Each “Tweet” is 200 characters max. Number of citations (SCOPUS): last check: 4 March 2022; in parentheses: number of citations per year. No. of subjects field specifies the number of T2DM subjects and possibly of other populations, if any. BIA: bioelectric impedance analysis; T2DM: type 2 diabetes; BMI: body mass index.

Ref. No.	“Tweet” on Study Characteristics/Outcomes	BIA Estimated/Calculated Parameters	No. of Subjects	Publication Year	No. of Citations
***Cross-sectional studies***					
*“Early” studies*					
[26]	Regional body composition analysis in T2DM patients shows decreased leg muscle mass, leg sarcopenia, and increased risk for cardiovascular diseases	Whole body and isolated (arms and legs) muscle mass (absolute and normalized to body weight), whole body fat mass (absolute and normalized)	198 T2DM, 198 healthy	2010	35 (2.9)
[27]	T2DM patients under bioelectrical impedance vector analysis show bioelectrical abnormalities, such as smaller appendicular muscular area, which can be risk factor for sarcopenia	Body resistance (R) and reactance (Xc), phase angle as arctan(Xc/R), and impedance vector as (R^2^ + Xc^2^)^0.5^	144 T2DM, 209 healthy	2013	10 (1.1)
[28]	In T2DM, DPP4 inhibitors treatment improves sarcopenic parameters as compared to sulphonylurea treatment	Fat-free mass (FFM), fat mass (FM), FFM/FM ratio, total, extracellular and intracellular water, skeletal muscle mass (SMM), SMM index	80 T2DM	2016	30 (5)
[29]	The skeletal muscle mass index is inversely associated with hepatic steatosis in T2DM men, likely due to factors such as insulin resistance, and abnormal levels of insulin-like growth factor 1	Skeletal muscle mass (normalized to total body weight), visceral fat area	145 T2DM	2016	47 (7.8)
[30]	In T2DM, there is direct correlation with BIA-derived parameters and plasma C-peptide, as well as inverse correlation with HDL-cholesterol, whereas no correlation is observed with glycemia and LDL	Body fat mass, total muscle mass, appendicular muscle mass, skeletal muscle index and percentage, total muscle index and percentage	359 T2DM	2017	1 (0.2)
[31]	The serum creatinine to cystatin C ratio (Cre/CysC) is usable as a simple screening tool to identify T2DM patients at high risk for sarcopenia, with an optimal cut-off value for Cre/CysC equal to 0.90	Appendicular skeletal muscle mass, skeletal muscle index	285 T2DM	2018	49 (12.3)
[32]	In obese T2DM, the prevalence of sarcopenia is low when diagnosed by the skeletal muscle index or the appendicular muscle mass/BMI ratio, and is much higher when using the body muscle ratio	Total fat mass, total muscle mass, sum of the appendicular muscle masses of the four limbs, skeletal muscle index, body muscle ratio	295 T2DM	2018	2 (0.5)
[33]	T2DM patients with visceral fat accumulation have low muscle quality, and patients with low muscle quality are more affected with cardiovascular diseases	Trunk, muscle masses of arms and legs, muscle quality (ratio of grip strength to arm muscle mass), skeletal muscle index	183 T2DM	2018	26 (6.5)
[34]	In elderly T2DM patients, sarcopenia is associated with blood pressure variability, but not with its absolute values	Body fat mass, skeletal muscle mass, appendicular muscle mass, skeletal muscle mass index (appendicular muscle mass /height squared)	146 T2DM	2018	20 (5)
[35]	Elderly T2DM patients are at higher risk for sarcopenia when having high body fat percentage but low BMI	Limb skeletal muscle mass, skeletal muscle mass index	267 T2DM	2019	35 (11.7)
[36]	Neuropathy screening questionnaire scores are higher in T2DM sarcopenic than in non-sarcopenic patients, thus a questionnaire may be used for screening for sarcopenia in subjects with diabetic neuropathy	Appendicular skeletal muscle mass (divided by height squared)	170 T2DM	2019	1 (0.3)
[37]	In sarcopenic obese patients, diabetic neuropathy prevalence reaches 96%, indicating a clear relationship between sarcopenia and diabetic neuropathy	Absolute skeletal muscle mass, skeletal muscle mass index	602 T2DM	2019	6 (2)
[38]	In T2DM, lower values of skeletal muscle mass normalized to visceral fat area (skeletal-to-visceral ratio) are associated with higher risks of developing non-alcoholic fatty liver disease	Lean body mass of arms and legs, appendicular skeletal muscle mass (sum of arms and legs lean masses), visceral fat area	445 T2DM	2019	5 (1.7)
*Recent studies*					
[39]	In hemodialysis patients, serum sclerostin is directly related to diabetes and inversely related to muscle mass	Fat-free mass, skeletal muscle mass index (fat-free mass divided by height squared)	41 T2DM, 51 non-diabetic	2020	8 (4)
[40]	In T2DM, low skeletal muscle mass, which is typical trait of sarcopenia, is independently associated with presence of carotid atherosclerosis	Skeletal muscle mass (SMM), skeletal muscle mass index (SMM divided by total body weight)	8202 T2DM	2020	8 (4)
[41]	In T2DM, sarcopenia appears significantly associated with impaired microcirculation, defined as low skin perfusion pressure	Appendicular skeletal muscle mass (as a sum of lean mass in the arms and legs) normalized to height squared	102 T2DM	2020	_ ^1^
[42]	In men with T2DM, sarcopenia appears independently associated with non-alcoholic fatty liver disease (NAFLD), suggesting sarcopenia as risk factor for NAFLD in that population	Appendicular skeletal muscle mass (ASM, as a sum of lean mass in the arms and legs), skeletal muscle mass index (ASM normalized to body weight)	4210 T2DM	2020	6 (3)
[43]	In T2DM, low extremity skeletal muscle mass may be a marker of possible co-occurring cognitive dysfunction	Skeletal muscle mass in legs and arms, appendicular lean mass (ASM, mass of four limbs), skeletal muscle mass index as ASM / height squared	1235 T2DM	2020	5 (2.5)
[44]	In T2DM, the prevalence of low muscle mass and sarcopenia may be found higher in older people and in people with normal BMI	Fat-free mass, body fat mass, percent body fat, visceral fat area, appendicular skeletal muscle mass (ASM), skeletal muscle index (ASM divided by height squared)	2404 T2DM	2021	1 (1)
[45]	In T2DM, some genetic factors (IRS1 and ADAMTSL3) contribute to interindividual variability in body composition, and this can help to establish effective methods for the prediction and prevention of sarcopenia	Total lean mass, appendicular lean mass, body fat mass, body resistance, skeletal muscle mass	176 T2DM	2021	0 (0)
[46]	Acarbose may contribute to decreased muscle mass and strength, thus muscle condition assessment and proper exercise may be important in T2DM patients using acarbose	Skeletal muscle mass (SMM), skeletal muscle (SMM divided by height squared)	1042 T2DM	2021	0 (0)
[47]	In T2DM elderly patients, the knee extension strength test can assist in the identification of probable and confirmed sarcopenia, as diagnosed by EWGSOP2 criteria	Fat mass, % body fat, total and segmental skeletal muscle mass (both legs, trunk, and both arms), appendicular skeletal mass index (sum of arms and legs masses/height squared)	100 T2DM	2022	_ ^1^
***Cross-sectional studied with nutritional data***					
[48]	Uncomplicated T2DM does not seem to accelerate age-related muscle mass or strength loss, thus aging may be more relevant than diabetes for sarcopenia risk	Fat-free mass (normalized to height squared)	32 T2DM, 34 non-diabetic	2014	11 (1.4)
[49]	In obese people with T2DM, preserved muscle fitness, especially of the lower extremities, may prevent sarcopenic obesity	Fat mass and lean mass, at five body segments (right and left upper extremities, trunk, right and left lower extremities)	26 T2DM	2015	15 (2.1)
[50]	Oxidative stress and antioxidant status may be associated with sarcopenia in T2DM elderly individuals; however, the association is likely mediated by other factors	Skeletal muscle mass, absolute skeletal muscle mass (normalizing to height squared)	60 T2DM	2019	4 (1.3)
[51]	In T2DM elderly patients, low energy intake is associated with sarcopenia	Skeletal muscle mass, appendicular muscle mass, body fat mass, skeletal muscle mass index (appendicular muscle mass/height squared)	391	2019	31 (10.3)
[52]	In T2DM non-obese patients without heart failure, brain natriuretic peptide levels are associated with sarcopenia	Skeletal muscle mass, appendicular muscle mass, body fat mass, skeletal muscle mass index (appendicular muscle mass/height squared)	433 T2DM	2019	8 (2.7)
[53]	In T2DM elderly patients, prevalence of sarcopenia is more than double when referring to the 2010 EWGSOP criteria, compared to revised 2019 criteria	Appendicular skeletal muscle mass (ASM, sum of arms and legs muscle mass), skeletal muscle mass index (ASM divided by height squared)	242 T2DM	2020	17 (8.5)
[54]	In T2DM elderly patients, omega-3 fatty acids contribute to increase muscle mass and improve skeletal muscle strength, thus decreasing sarcopenia risk	Skeletal muscle mass, appendicular muscle mass, body fat mass, skeletal muscle mass index (appendicular muscle mass/height squared)	342 T2DM	2020	7 (3.5)
[55]	In T2DM, low irisin levels and poor glycemic control are independent risk factors for sarcopenia, and especially for sarcopenic obesity	Fat mass, fat-free mass, appendicular skeletal muscle (ASM), skeletal muscle index (ASM/height squared)	90 T2DM	2021	4 (4)
***Longitudinal (interventional) studies***					
[56]	In T2DM, treatment with dapagliflozin for six months improves glycemic control and reduced body weight without reducing muscle mass	Total fat mass, soft lean mass, skeletal muscle mass at five body segments (arms, legs, trunk), skeletal muscle mass index (normalization to height squared)	50 T2DM	2018	36 (9)
[57]	In T2DM, reducing loss of fat-free mass over time may reduce insulin resistance and prediabetes risk, particularly among individuals with overweight/obesity	Fat mass, fat-free mass (FFM), relative FFM (normalization to body weight), relative FFM percent change between baseline and follow-up	6264 T2DM	2021	3 (3)
[58]	In T2DM patients, low baseline skeletal muscle mass and its reduction over time is associated with increased risk for progression of chronic kidney disease	Total skeletal muscle mass, skeletal muscle mass index (normalization to weight)	1272 T2DM	2021	2 (2)
[59]	Elderly women with low skeletal muscle or overt sarcopenia have higher probability of developing glucose intolerance or even diabetes	Body resistance, reactance, phase angle, fat mass appendicular skeletal muscle mass	159 non-T2DM (at baseline)	2021	3 (3)
[60]	T2DM and chronic kidney disease are synergically associated with muscle mass loss over time, and mortality is higher in individuals with muscle loss	Fat-free mass index (details not provided)	6247 subjects (some with T2DM)	2021	0 (0)
[61]	Sarcopenia with co-existent T2DM was associated with increased risk for readmission and infections among hospitalized cardiac patients	Fat mass, appendicular skeletal muscle mass, appendicular skeletal muscle mass index (normalization to height squared)	50 T2DM, 50 non-T2DM	2021	0 (0)
[62]	In T2DM elderly people with long diabetes duration, low muscle strength and insulin resistance are the main risk factors for aggravated glycemic control	Muscle mass, fat mass (both normalized to weight, and stratified in quartiles)	100 T2DM	2021	0 (0)
[63]	COVID-19 pandemic restrictions cause muscle mass loss in older patents with T2DM; thus, exercise and adequate diet intake are needed to prevent sarcopenia	Appendicular muscle mass, fat mass, skeletal muscle mass index (SMI, as appendicular muscle mass/height squared), percent fat mass (fat mass/body weight), change in SMI per year	56 T2DM	2021	2 (1)
[64]	In T2DM, branched-chain amino acids (valine, leucine and isoleucine) appear to have preventive role in muscle mass loss	Skeletal muscle mass, skeletal muscle mass index (normalization to weight)	1140 T2DM	2022	0 (0)
[65]	Hemodialysis patients with T2DM show overnutrition, but also paradoxically higher predisposition to protein–energy wasting (possible traits of obese sarcopenia)	Lean tissue mass and fat tissue mass, lean tissue index and fat tissue index (normalization to height squared), overhydration and relative overhydration	198 T2DM, 317 non-T2DM	2022	_ ^1^

^1^ Not reported in SCOPUS (at the indicated date of last check).

## Data Availability

Not applicable.
